# Significance of β-Galactoside α2,6 Sialyltranferase 1 in Cancers

**DOI:** 10.3390/molecules20057509

**Published:** 2015-04-24

**Authors:** Jishun Lu, Jianguo Gu

**Affiliations:** Division of Regulatory Glycobiology, Institute of Molecular Biomembrane and Glycobiology, Tohoku Pharmaceutical University, 4-4-1 Komatsushima, Aobaku, Sendai, Miyagi 981-8558, Japan

**Keywords:** cancer metastasis, sialylation, ST6Gal I

## Abstract

Altered glycosylation is a common feature of cancer cells. It takes a variety of forms, which includes loss of expression or excessive expression of some structures, the accumulation of precursors, the appearance of novel structures, *etc*. Notably, these changes in glycan structure do not occur as a random consequence of disorder biology. Only a limited subset of oligosaccharides is found frequently enriched on the tumor cell surface and implicated in different tumor phenotypes. Among these, altered sialylation has long been associated with metastatic cell behaviors such as invasion and enhanced cell survival and accumulating evidence points to the alteration occurring in the sialic acid linkage to other sugars, which normally exists in three main configurations: α2,3, α2,6, and α2,8, catalyzed by a group of sialyltransferases. The aberrant expression of all three configurations has been described in cancer progression. However, the increased α2,6 sialylation catalyzed by β-galactoside α2,6 sialyltranferase 1 (ST6Gal I) is frequently observed in many types of the cancers. In this review, we describe the findings on the role of ST6Gal I in cancer progression, and highlight in particular the knowledge of how ST6Gal I-mediated α2,6 sialylated glycans or sialylated carrier proteins regulate cell signaling to promote the malignant phenotype of human carcinoma.

## 1. Introduction

It has long been known that cell surface glycans undergo dramatic changes upon carcinogenesis. During the past decades, a large number of studies have demonstrated the importance of these carbohydrates in tumor progression and deepened our understanding of the molecular mechanisms linking altered glycosylation to tumor behavior. Many reviews have discussed the roles of different tumor-associated glycans in different stages of human cancer progression [1,2,3,4]. Among these, altered sialylation on cell surface is always a part that could not be ignored. Sialic acids are a diverse group of negatively charged monosaccharides typically found as terminal components attached to cell surface glycoconjugates including *N*-glycans, *O*-glycans and glycosphingolipids [5,6]. Sialic acids have been primarily described in mammals, however they are also found in lower vertebrates and in invertebrates [7,8]. Sialic acids comprise more than 50 naturally occurring derivatives of the nine-carbon sugar neuraminic acid, which represent their first level of diversity [9]. In mammalian cells, the most common sialic acids are *i*-acetylneuraminic acid (Neu5Ac) and *N*-glycolylneuraminic acid (Neu5Gc), although only the former is present in human cells [10]. The second level of diversity arises from the sialic acid linkage to sugar chains. To date, sialic acids are known to be linked via an α2,3 or α2,6 bond to Gal/GalNAc, or α2,8 bond to sialic acid in proteins through a group of sialyltransferases. Given the relatively strong electronegative charge of sialic acids and their location at the outmost reaches of the cell surface, it is not surprising that sialic acids modulate the conformation and stabilization of molecules and membranes, interactions with the environment, as well as normal processes including transmembrane signaling, fertilization, growth, differentiation and apoptosis [11,12]. On the other hand, altered sialylation has long been associated with the malignancy of carcinoma. High expression of sialic acids has been proposed to protect cancer cells from recognition and eradication by the immune system [13]. However, there is limited information regarding the molecular details of how distinct sialylated structures or sialylated carrier proteins regulate cell signaling to control metastatic cell behaviors including invasion and enhanced cell survival. Many cancer associated sialylated structures, which includes sialyl Thomsen-nouvelle antigen (sialyl Tn), sialyl Lewis antigen (sLe), α2,6 sialylated lactosamine, polysialic acid and gangliosides have been identified and their potential functions are well documented by some reviews and books [11,14,15,16,17]. Altered expression of these structures in cancer cells could result from multiple mechanisms. Loss of expression or excessive expression of certain sialyltransferases is frequently observed. Table 1 shows the sialyltransferases identified in human and most of them have been reported to express abnormally in cancers and are proposed to contribute to cancer progression [16]. The roles of different sialyltransferases in tumor progression have been comprehensively described in several reviews [18,19]. Here, we are focusing on the β-galactoside α2,6 sialyltranferase 1 (ST6Gal I), an enzyme catalyzing the α2,6 sialylation on *N*-glycans, because the altered expression of ST6Gal I are observed in many types of cancers (Table 1) and increasing evidence indicates its fundamental roles in tumor malignancy. In detail, we describe the recent findings on ST6Gal I in cancer progression, where the mechanistic roles of ST6Gal I in tumor malignant progression are highlighted and the mechanisms governing the cell surface α2,6 sialylation are discussed.

**Table 1:** Cloned sialyltransferases and their involvement in human cancers.

Sialyltransferases	Acceptor Sequence(s)/(Carrier Type)	Types of Cancers in Which the Altered Expression Observed	References **
ST3Gal I	Galβ1-3GalNAc *, Galβ1-3GlcNAc/(*O*-glycans, glycolipids)	Breast, bladder, colon	[20,21,22]
ST3Gal II	Galβ1-3GalNAc/(*O*-glycans, glycolipids)	Prostate, colon	[22,23]
ST3Gal III	Galβ1-3*/4GlcNAc(*N*-glycans, *O*-glycans, glycolipids)	Stomach, pancreas, extrahepatic bile duct, cervix	[24,25,26,27]
ST3Gal IV	Galβ1-3GalNAc, Galβ1-3/4*GlcNAc/(*N*-glycans, *O*-glycans, glycolipids)	Renal cell, stomach	[28,29]
ST3Gal V	Galβ1-4Glc/(glycolipids)	Pediatric leukemia	[30]
ST3Gal VI	Galβ1-3/4*GlcNAc/(*N*-glycans, *O*-glycans, glycolipids)		
ST6Gal I	Galβ1-4GlcNAc/(*N*-glycans)	Colon, breast, cervix, choriocarcinomas, acute myeloid leukemias, liver, brain	[18,31,32,33,34,35,36,37,38,39,40]
ST6Gal II	Galβ1-4GlcNAc/(*N*-glycans)		
ST6GalNAc I	Galβ1-3GalNAc/(*O*-glycans)	Stomach, pancreas, colon, ovary, breast	[15,41,42,43,44]
ST6GalNAc II	Galβ1-3GalNAc/(*O*-glycans)	Colon	[42,45]
ST6GalNAc III	Galβ1-3GalNAc, G_M1b_/(*O*-glycans, glycolipids)		
ST6GalNAc IV	Galβ1-3GalNAc, G_M1b_/(*O*-glycans, glycolipids)		
ST6GalNAc V	G_M1b_/(glycolipids)	Colon, breast	[46,47]
ST6GalNAc VI	G_M1b_, G_T1b_/(glycolipids)	Colon	[48]
ST8Sia I	G_M3_/(glycolipids)	Breast cancer, pediatric acute leukemia	[30,49]
ST8Sia II	Sia2-3/6/8Galβ1-4GlcNAc/(*N*-glycans)	Liver	[37]
ST8Sia III	G_T3_, Siaα2-3Galβ1-4GlcNAc/(*N*-glycans, glycolipids)	Glioblastoma	[50]
ST8Sia IV	Sia2-3/6/8Galβ1-4GlcNAc/(*N*-glycans)		
ST8Sia V	G_D3_, G_M1b_ G_D1a_, G_T1b_, G_Q1c_/(glycolipids)		

* The preferred acceptor sequence; ** The authors apologize to many researchers, whose outstanding papers are not cited here due to a space limitation.

## 2. The Structure of ST6Gal I Gene and Protein as well as the Lectins Specifically Recognizing α2,6 Sialylated Sugar Chains

Among the sialyltransferases identified, ST6Gal I is the first to be cloned and biochemical, genetic studies have continued to use this enzyme as a paradigm for understanding Golgi glycosylation [51]. The initial report on the structure of ST6Gal I gene and protein is derived from rat liver [51]. It has been shown that this sialyltransferase comprise 403 residues coded by approximately 4.7 kb mRNA and the topology of the enzyme in the Golgi apparatus consists of a short NH2-terminal cytoplasmic domain, a 17-residue hydrophobic sequence which serves as the membrane anchor and signal sequence, and a large luminal, catalytic domain. Further study showed that the rat ST6Gal I gene produces three different sized mRNA through alternative splicing and promoter utilization in tissue-specific fashion [52]. Similarly, this tissue-specific fashion is also observed in human ST6Gal I transcripts [53,54]. Three major mRNA species have been identified, which share a common protein coding region but diverge in the 5'-untranslated regions. The first cloned from a placenta cDNA library contains the 5'-untranslated exons Y and Z (Y+Z form) [55]. The second lacks exons Y and Z but contains 5'-untranslated exon X [56]. The third species initially characterized from HepG2 hepatoma cells lacks Y, Z and X. Instead, it contains a short specific sequence in front of exon I [57]. The different transcripts of ST6Gal I have been shown to result from the regulation of its multiple and distinct promoter regions [58]. Although the regulatory mechanism remains unclear, this strategy provides a reasonable explanation for the observation that most tissues express ST6Gal1 in humans, but the level of expression varies dramatically. Under pathological conditions, cells seems to have different preference for ST6Gal I transcripts. Both the Y+Z form and hepatic transcripts were detectable in normal and cancer tissues of colon but that latter form had a marked tendency to accumulate in cancer [58]. The regulation of the different promoter of ST6Gal I will be discussed later in this review.

The study of biological functions of ST6Gal I mediated α2,6-sialylation under physiological and pathological conditions are facilitated with the discovery of lectins which specifically recognize the Sia(α2-6)Gal/GalNAc sequence. The earliest identified is the lectin named Sambucus nigra agglutinin (SNA) [51,59]. The level of α2,6 sialylation of cell glycoproteins determined by SNA lectin has been demonstrated to closely correlate with the level of ST6Gal I enzyme activity in colon cancer cell lines, and the colon cancer tissues with the high expression ST6Gal I present a high reactivity to SNA [38,54]. In addition to SNA, another lectin Trichosanthes japonica agglutinin I (TJA-I) has also been reported to specifically recognize the Sia(α2-6)Gal/GalNAc residues [60]. Histochemical study with TJA-I lectin showed that normal mucosa and benign adenoma tissues from the patients with colonic adenocarcinomas were not stained and 83% of well and moderately differentiated colon adenocarcinomas reacted with this lectin, indicating a potential application of TJA-I staining for early diagnosis of colon cancers [61]. Both of these lectins have been widely utilized and become useful and convenient tools for the indication of the α2,6-sialylation level.

## 3. Functions of ST6Gal I in Cancer Progression

The up-regulated expression of ST6Gal I was first described in colon cancer, but successively confirmed in other carcinomas of breast, liver, cervix, choriocarcinomas, acute myeloid leukemias and some malignancies of the brain as well [18,31,32,33,34,35,36,37,38,39,40]. The α2,6 sialylated blood group type 2H catalyzed by ST6Gal I in colon cancer has been reported to be predictive markers of poor prognosis [62]. It is worth mentioning that altered epression of ST6Gal I is observed in hepatocarcimoma, but not the cirrhosis [40]. All this information suggests that ST6Gal I plays important roles in tumor progression. So, how does this enzyme benefit the tumor cells? The hallmark of early carcinogenesis is the acquisition of a highly proliferative activity by the transforming cells. However, ST6Gal I seems to have no effect on it, because quantitative lectin-histochemical and immune-histochemical studies on the occurrence of α2,3 and α2,6 sialic acid residues in colorectal carcinomas showed that 2,6 sialylated glycoconjugates did not display any association with local tumor growth, while α2,3 sialylation positively correlated with tumor growth and significantly increased at tumor Stage I and Stage II, but decreased in advanced carcinomas [63]. Consistent with the observation in colon cancer, increased ST6Gal I expression in breast cancer was observed only by a group of patients mainly of Stage III [31]. A study from Varki’s group showed that mammary tumors developed by PyMT mice in a ST6Gal I null background displayed increased differentiation but the same growth rate as those grown in the PyMT mice expressing ST6Gal I [64]. However, paradoxically, overexpression of ST6Gal I in colon cancer cells has also been reported to reduce tumorigenicity [65] and conversely, inhibition of ST6Gal I expression increased cell proliferation and tumor growth *in vitro* and *in vivo* [66]. Given that ST6Gal I and α2,3 sialyltransferases share common substrates and overexpression of ST6Gal I could lead to the decrease in α2,3 sialylation through substrate competition mechanisms [67], it is reasonable to hypothesize that the inhibitory effects of ST6Gal I overexpression on tumor growth is the direct result of decreased α2,3 sialylation.

In contrast to the elusive role in the tumor growth, increasing evidence indicates that ST6Gal I is critical for the tumor malignancy including metastasis and invasion. It has been shown in colon cancer that metastatic tumor growth is accompanied by a significant increase of α2,6 sialylated carbohydrate sequences produced by ST6Gal I [63]. Knockdown of ST6Gal I significantly inhibits the cell metastasis in diverse carcinomas [68,69,70]. Conversely, forced expression of ST6Gal I in MDA-MB-435 human mammary tumor cells and OV4 ovarian carcinoma cells leads to reduced cell–cell adhesion and enhanced capacity for invasion [69,71]. Animal models also implicate ST6Gal I in tumor metastasis. Neuraminidase treatment of metastatic murine cell lines dramatically decreases the amount of liver metastasis after splenic injection [72]. Despite the well-established link between ST6Gal I and cell migration, the underlying mechanisms remain unclear. Several groups including us recently demonstrate that ST6Gal I promotes the cell motility by activating the PI3K/Akt signaling pathway [37,73]. Also, *in vitro* studies show that the effects of ST6Gal I on cell migratory response are mediated, at least in part, by the α2,6 sialylation of the β1 integrin because cells deficient in β1 integrin do not exhibit differential invasion upon forced expression of ST6Gal I expression [71,74,75].

**Figure 1:**
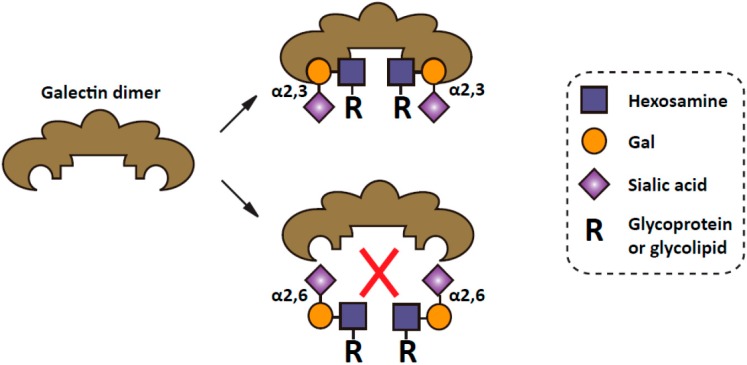
α2,6 sialylation inhibits the galectin binding to the carbohydrate [11]. The free hydroxyl group on the six carbon of galactose is required for the binding to galectins [83]. The addition of α2,6 linked sialic acids at this site by sialyltransferases, therefore, could block their interaction. In contrast, α2,3 sialic acids have little effects on galectin binding.

On the other hand, the selective enrichment of α2,6 sialic acids produced by ST6Gal I on tumor cells has also been shown to render the cells resistant to apoptosis. One striking example is the effect of α2,6 sialylation on the apoptosis signaling mediated by cell galectins. Galectins are a family of animal lectins with affinity for β-galactosides [76,77,78]. A number of galectins have been shown to interact with cell-surface and extracellular matrix glycoconjugates through lectin–carbohydrate interactions. Through this action, some galectins are capable of inducing apoptosis [79,80,81]. However, recent study shows that α2,6 sialylation of galactose serves as a generic inhibitor of galectin binding and upregulation of cell surface α2,6 sialylation is able to block the binding of pro-apoptotic galectins, thereby promoting tumor cell survival [82,83,84]. In this regard, it is worth mentioning that the anti-apoptotic effect of α2,6 sialylation is specific as compared with α2,3 sialylation in that, unlike α2,6 sialic acids, α2,3 sialic acids have little effects on galectin binding (Figure 1). In addition to the galectin-mediated pathway, it has also been shown that α2,6 sialylation could elicit its anti-apoptotic effects through inhibiting the cell death pathways initiated by Fas and TNFR1 [85,86]. Reduced Fas-mediated apoptosis is a well-established factor in tumor survival and Fas expression is down-regulated in many different tumor types [87,88,89]. However, some cells express high levels of Fas, but are yet resistant to Fas-induced apoptosis [90,91,92]. In fact, it has been shown that Fas pro-apoptotic activity is masked by sialylation [93,94,95]. Recent work clearly defines that α2,6 sialic acid linkage is functionally important, as α2,6 sialylation of Fas (but not α2,3 sialylation) could inhibit Fas apoptotic activity by interfering the formation of death inducing signaling complex and restraining Fas receptor internalization [85]. Similar to Fas, ST6Gal I mediated α2,6 sialylation of TNFR death receptor blocks apoptosis directed by the TNFR1 ligand, TNFα [86]. Consistent with the inhibitory effect of α2,6 sialylation on apoptosis through galectins, Fas and TNFR1, upregulation of ST6Gal I was reported to confer radiation resistance in colon cancer cell lines, as well as multidrug resistance in human acute myeloid leukemia [96,97].

Cancer stem cells (CSCs) refer to a minority population of cancer cells that are capable of self-renewal and generation of differentiated progeny. Eradication of this rare population is a new insight in cancer treatment. Intriguingly, high expression of ST6Gal I has been correlated with human induced pluripotent stem cells and CSCs, indicating that ST6Gal I activity may be involved in maintaining some aspect of stem like cell behavior [98]. Considering the fact that tumors induced in ST6Gal I knockout mice were more differentiated compared with those in the wild type background [64], it is postulated that ST6Gal I could be important for an immature or undifferentiated cell phenotype. On the other hand, epithelial-mesenchymal transition (EMT) has been regarded as one mechanism for the generation of CSCs. EMT describes a trans-differentiation process that allows fully polarized epithelial cells to undergo multiple biochemical changes, enabling them to acquire a mesenchymal identity with properties of stem-like cells. Recently, our group showed that ST6Gal I expression was required for the TGF-β induced EMT [99]. Knockdown of ST6Gal I prevented TGF-β-induced increase in cell migration. Therefore, ST6Gal I in CSCs is highly likely to contribute to maintaining the metastatic property as well. In addition, as mentioned above, a number of reports show that ST6Gal I confers resistance to apoptosis. Thus, we could not exclude the possibility that expression of ST6Gal I reduces the apoptosis sensitivity in response to various stimuli, thereby extending the cell lifespan of the CSCs.

The functions of ST6Gal I in cancer progression could be more complicated than discussed above. The pro-migratory and anti-apoptotic roles have been challenged by several reports that: (1) forced expression of ST6Gal I suppressed the cell migration and enhanced the cell death induced by chemotherapeutic agents in glioma cells [67,100]; (2) ST6Gal I loss was associated with increasing invasiveness in bladder carcinogenesis [101]. In glioma cells, it was shown that instead of ST6Gal I, ST3Gal IV contributed to the cell migration and survival and overexpression of ST6Gal I could suppress the ST3Gal IV mediated α2,3 sialylation by competing for their common substrates. Whether a similar mechanism occurs in bladder carcinomas has to be confirmed. On the other hand, a recent study directly demonstrated that ST6Gal I is also involved in tumor angiogenesis. Increased ST6Gal I mediated sialylation suppressed VEGF-independent angiogenesis in tumor growth by preventing Gal1 binding and elimination of α2,6 linked sialic acids conferred resistance to anti-vascular endothelial growth factors-targeted treatment [102].

## 4. Mechanistic Roles of ST6Gal I in Cancer Progression

While not completely understood, the functions of α2,6 sialylation described above may be exerted by affecting the structures of attached glycans or carrier proteins. In the first case, as mentioned earlier, the addition of α2,6 sialic acid to the terminal galactose of *N*-glycans masks the galectin recognition sites for binding of β-galactoses, which in turn switches off the galectin functions including adhesion, migration and apoptosis. In contrast to the inhibitory effect on the glycan–galectin binding, α2,6 sialic acids have also been reported to bind specifically to the siglec-2 family of lectins [103]. Since siglec-2 are mainly expressed by immune cells, the potential functions of siglecs in tumor biology has been envisioned that changes in tumor cell sialylation could affect the activity of siglec-expressing immune cells, and consequently modulate the anti-tumor immune response. Clearly, further evidence is needed for this hypothesis. On the other hand, α2,6 sialic acids have direct effects on the structure/function of specific sialylated glycoproteins. α2,6 sialylation has been shown to alter conformation of the β1 integrin [104], clustering of the CD45 [105], EGFR [106] and PECAM [107], cell surface retention of PECAM [107] and Fas death receptor [85]. There is also evidence that α2,6 sialylation of galectin receptors causes release from the galectin lattice, leading to receptor internalization [108]. Given the relatively large size and negative charge of sialic acids, these findings should not be surprising. Taken together, abundant literature indicates that α2,6 sialylation holds potential to influence tumor cell behaviors through many different mechanisms.

## 5. Regulatory Mechanisms of ST6Gal I Expression in Cancer Progression

**Figure 2:**
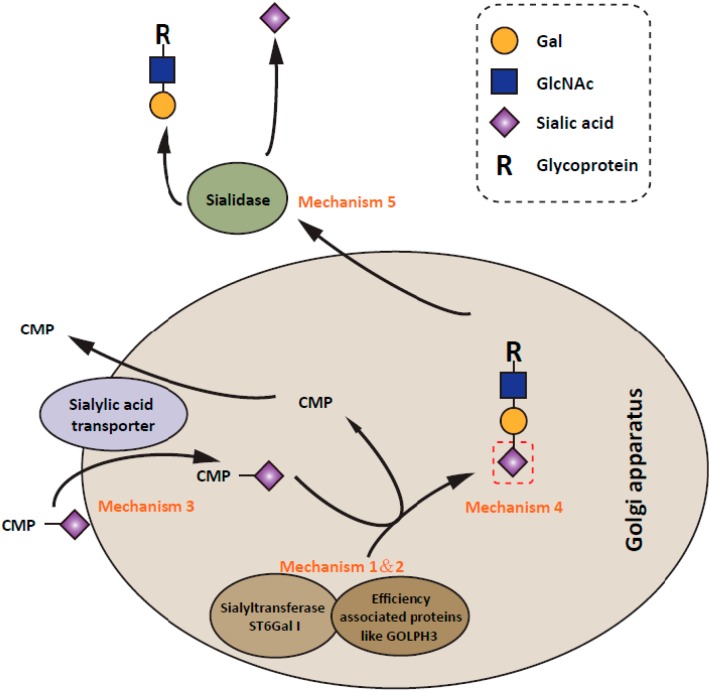
Schematic representation of the regulation of α2,6 sialylation expression. The expression level of surface α2,6 sialylation is increased in tumors by several mechanisms. (1) Most commonly, ST6Gal I transcription is regulated by some transcription factors and methylation modification [18,31,32,33,34,35,36,37,101,130]; (2) Some factors like GOLPH3, recently, has been shown to interact with ST6Gal I, thereby modulating the efficiency of the sialylation [73]. (3) In addition, the expression levels of sialic acid transporter could affect α2,6 sialylation expression by regulating the donor substrate reservoir of ST6Gal I [118,119]; (4) The distribution as well as the amount of α2,6 sialic acids on cell surface also depend on the expression pattern of oligosaccharide acceptors [127,128,129]; (5) Further, as observed in many types of tumors, down-regulation of sialidases is usually accompanied by the upregulation of α2,6 sialylation expression [120,121,122,123,124].

Given the accumulating evidence for the importance of ST6Gal I mediated α2,6 sialylation in cancer progression, much attention has been paid to elucidating the regulatory mechanisms of its expression. It has been shown that the expression of α2,6 sialylation on tumor cell surface can be modulated at different levels (Figure 2). The most frequently observed is the modulation of the ST6Gal I transcription. ST6Gal I expression is positively regulated by oncogenic N-ras and H-ras and negatively regulated by the tumor suppressor transcription factor RUN3 [109,110,111,112,113]. On the other hand, caveolin-1, a gene with a prevalent tumor suppressor activity, has been reported to stimulate ST6Gal transcription [114]. In spite of the fact that many proteins have been reportedly involved in the modulation of ST6Gal I transcription, the information regarding how ST6Gal I is transcriptionally regulated still remains obscure. Expression of ST6Gal I has been shown to be regulated by different promoters (designated as P1, P2 and P3) in different cancers [115]. The activity of P1 promoter is specifically enhanced in cervical cancer tissue [27,116]. Further analysis of P1 promoter by luciferase assays in cervical and hepatic cell lines showed that mutation of Sp1 or HNF1 binding sites affected the promoter activity only in HepG2 cell line, but not in C33A cells, indicating that the regulation of ST6Gal I promoter activity is cell type specific [117]. Moreover, in contrast to the enhanced activity of P1 promoter in cervical cancer, ST6Gal I expression is induced by Ras oncogene in NIH3T3 cells via its P3 promoter [112] which suggests that the transcription of ST6Gal I is regulated by different mechanisms under different biological scenarios. In addition to transcription factors, the promoter activity of ST6Gal I is also regulated by the epigenetic modification [101]. ST6Gal I promoter methylation resulted in ST6gal I gene silencing in human bladder cancer. Beyond its promoter activity, ST6Gal1 expression could be regulated at a post-transcriptional level. Our recent report showed that ST6Gal I formed a complex with GOLPH3, an oncogene that functions in secretory trafficking at the Golgi. Knockdown of this gene in MDA-MB-231 breast cancer cells led to the down-regulation of both α2,3 sialylation and α2,6 sialylation, but had no effect on the transcription of sialyltransferases [73]. Further, alternative pathways could also regulate the α2,6 sialylation on the cell surface. For instance, although not a human cell line, CHO-Lec2 cells which are unable to transport CMP-sialic acid (substrate for sialyltranferases) into the Golgi vesicle, have a 70%–90% deficiency in sialic acids on the glycoproteins and gangliosides [118,119]. In addition, several sialidases in tumor cells are expressed abnormally and act at the cell surface [120,121,122,123,124]. A reduction of certain sialidases like Neu1, which removes sialic acid residues on oligosaccharides leads to higher levels of cell surface sialylation of human cancers [124]. Moreover, different sialyltransferases have different acceptor preferences. Recent structure studies of ST6Gal I and ST3GAL1 (which catalyzes 2,3 sialylation on *O*-glycans) clearly demonstrated the difference in their glycan acceptor binding domain [125,126]. Therefore, it is reasonable to hypothesize that the expression level as well as the distribution of α2,3 and α2,6 sialylation could be also modulated by the expression pattern of oligosaccharides that sialyltransferases catalyze. Consistent with this idea, it was shown that biantennary N-glycan could be easily sialylated by ST6Gal I but not α2,3 sialyltransferases and a higher degree of branching of the acceptors led to a decrease in the rate of sialylation [127,128]. Also, knockout of GnT-V, an enzyme catalyzes the α1,6 branched GlcNAc structure of N-glycan, led to increased α2,6 sialylation and decreased α2,3 sialylation [129]. Better understanding of the substrate specificity of ST6Gal I may contribute to the development of ST6Gal I-specific inhibitory drug.

## 6. Conclusions and Future Directions

Given increasing evidence implicating ST6Gal I in multiple cancers, much effort has been undertaken to clarify the functions of this enzyme in cancer progression during the last decade. Furthermore, it is becoming clear that ST6Gal I plays important roles in regulating tumor metastatic behaviors including migration and enhanced cell survival. In contrast, there is a marked dearth of information on how tumor cells regulate the expression of ST6Gal I and α2,6 sialylation. Defining regulatory mechanisms and specific ST6Gal I substrates will be necessary for a complete understanding of the roles of ST6Gal I during cancer progression and may provide new insights for targeting aggressiveness and drug resistance of the cancer cell by manipulating the level of cell surface α2,6 sialylation. In addition, although ST6Gal I is mainly localized in the Golgi apparatus, this protein can also be proteolytically processed to a soluble form and secreted into the serum [51,131]. The serum ST6Gal I is involved in the generation of the cell-surface carbohydrate determinants and differentiation antigens HB-6, CD75, and CD76 [132]. Its enzymatic activity in serum is elevated during the hepatic inflammatory response, a cumulative homeostatic process executed in response to tissue injury, trauma, infection, or tumor burden [133]. Therefore, there is a critical need for elucidating the roles of soluble ST6Gal I in physiological and pathological processes.
